# Association of neurite orientation dispersion and density imaging with cognitive impairment in nasopharyngeal carcinoma patients with radiation-induced temporal lobe injury

**DOI:** 10.3389/fnhum.2026.1809205

**Published:** 2026-06-01

**Authors:** Weike Zeng, Qin Wen, Yun Su, Qingxia Peng, Zhuoheng Yan, Xinyin Chen, Xu Yan, Haodong Qin, Jianing Li, Jiaji Mao

**Affiliations:** 1 Department of Radiology, Sun Yat-Sen Memorial Hospital, Sun Yat-Sen University, Guangzhou, China; 2 Department of Neurology, Sun Yat-Sen Memorial Hospital, Sun Yat-Sen University, Guangzhou, China; 3 MR Research Collaboration Team, Diagnostic Imaging, Siemens Healthineers Ltd., Shanghai, China

**Keywords:** cognitive impairment, diffusion-weighted imaging, nasopharyngeal carcinoma, neurite orientation dispersion and density imaging, radiation-induced temporal lobe injury

## Abstract

**Background:**

To evaluate whether neurite orientation dispersion and density imaging (NODDI) can reflect cognitive impairment (CI) in nasopharyngeal carcinoma (NPC) patients with late-delayed radiation-induced temporal lobe injury (RTLI).

**Methods:**

In this prospective study, 90 NPC patients with RTLI underwent structural MRI, conventional diffusion-weighted imaging, and NODDI. Cognitive function was assessed using the Montreal Cognitive Assessment (MoCA; score < 26 indicated CI). NODDI-derived parameters—intra-neurite volume fraction (ICVF), isotropic volume fraction (ISOVF), and orientation dispersion index (ODI)—and apparent diffusion coefficient (ADC) were compared between patients with and without CI. Diagnostic performance and correlations with MoCA scores were analyzed.

**Results:**

Among 90 patients, 66 had CI. CI patients exhibited significantly lower ICVF and ODI (*p* < 0.001, *p* = 0.047, respectively), while ISOVF and ADC did not differ (*p* > 0.05). ICVF demonstrated superior discriminative performance (AUC = 0.869) versus ODI (AUC = 0.638). Correlation analyses demonstrated a strong positive association between ICVF and the MoCA scores (*ρ* = 0.781, *p* < 0.001), a weak negative correlation for ISOVF (*ρ* = −0.310, *p* = 0.003) and ADC (*ρ* = −0.215, *p* = 0.041), and no significant correlations for ODI.

**Conclusion:**

NODDI-derived ICVF is associated with CI and correlates with MoCA scores in NPC patients with RTLI.

## 1 Introduction

Arising from the nasopharyngeal epithelium, nasopharyngeal carcinoma (NPC) is characterized by a distinctly uneven geographical prevalence as a malignant tumor. This disease continues to pose a major challenge to public health systems, especially across regions of East and Southeast Asia (Bray et al., 2024). Radiotherapy is commonly the primary treatment modality for nasopharyngeal carcinoma (NPC). This approach is favored due to both the anatomical intricacy of the tumor site and the notable sensitivity of NPC to radiation (Beddok et al., 2020; Wong et al., 2021). Due to the vicinity of crucial organs surrounding the nasopharynx, the temporal lobe frequently receives unavoidable incidental radiation exposure (Pfister et al., 2020). Consequently, radiation-induced temporal lobe injury (RTLI) stands as one of the most serious delayed adverse effects following radiotherapy, with reported incidence rates ranging from 5 to 30% within ten years (Bao et al., 2022; Jlailati et al., 2025).

Late-delayed RTLI is clinically defined as damage emerging six months to multiple years post-radiotherapy and represents the most prevalent and severe form of RTLI (Guo et al., 2018). This form of brain injury follows a progressive and frequently relapsing course, leading to largely irreversible functional and cognitive deficits (Wu et al., 2020). Clinically, cognitive impairment (CI) manifests as severe symptoms, including altered consciousness, memory loss, executive dysfunction, and apraxia, all of which profoundly compromise quality of life (Guan et al., 2023). Current management remains primarily symptomatic, relying on glucocorticoids, anti-vascular endothelial growth factor agents, and neuroprotective drugs to alleviate symptoms and slow functional decline. Therefore, accurate detection of CI is essential for initiating medical interventions, adjusting drug dosages, and optimizing therapeutic outcomes (Turnquist et al., 2020; Li and Ding, 2024). While cognitive assessment tools such as the Montreal Cognitive Assessment (MoCA) have been frequently employed for diagnostic purposes (Jia et al., 2021), they are limited by the requirement for time-consuming assessments by experienced specialists and potential confounding from the patient’s test-taking state, such as fatigue or attention (Rose et al., 2021). These limitations underscore the need for more objective and efficient imaging biomarkers to reflect CI in patients with late-delayed RTLI.

Magnetic resonance imaging serves as the principal diagnostic tool for identifying late-delayed RTLI. Structural MRI demonstrates a spectrum of evolving features, ranging from initial finger-like hyperintensities on T2-weighted imaging to characteristic “Swiss cheese” or “soap bubble” enhancement patterns, and finally to late cyst-like formations (Lee et al., 1988; Lee et al., 1990). Critically, these radiological signs exhibit no direct correlation with clinical symptom severity. Consequently, structural MRI cannot accurately evaluate cognitive function in these patients. Diffusion magnetic resonance imaging (dMRI) extends beyond structural MRI by quantifying the random motion of water molecules, thereby providing superior sensitivity to microstructural tissue properties (Shamir and Assaf, 2025). The most widely used dMRI technique, DWI, is analyzed using the apparent diffusion coefficient (ADC). This metric quantifies the general extent of water molecule movement within tissues. However, DWI studies investigating RTLI have reported conflicting ADC findings in the temporal lobe post-radiotherapy, with values ranging from marked reductions to non-significant alterations (Wang et al., 2012; Huang et al., 2025). This highlights the essential requirement for more advanced imaging biomarkers to accurately characterize microstructural damage in the temporal lobe and its correlation with CI.

Recently, the three-compartment biophysical model known as neurite orientation dispersion and density imaging (NODDI) has emerged as a powerful dMRI technique for characterizing the complexity and heterogeneity of brain microstructure (Palacios et al., 2020; Vogt et al., 2022). This model quantifies brain tissue microstructure by estimating three key parameters: the intra-cellular volume fraction (ICVF), reflecting neurite density; the orientation dispersion index (ODI), representing the degree of neurite orientation coherence; and the isotropic volume fraction (ISOVF), indicating the proportion of free water (Zhang et al., 2012). Given that cognitive function relies on the structural integrity and organization of neurites, NODDI-derived parameters hold promise as sensitive biomarkers for evaluating cognitive changes (Zhang et al., 2012). Indeed, NODDI has proven valuable in detecting microstructural changes of the brain and assessing cognitive function in diseases such as Alzheimer’s disease (Cheng et al., 2024), orthostatic hypotension (Cheng et al., 2024), and benzodiazepine use disorders (Yi et al., 2025). However, it remains unknown whether NODDI metrics are associated with CI in patients with late-delayed RTLI. Therefore, this study aims to evaluate the potential of NODDI-derived metrics compared with conventional DWI-derived ADC values for differentiating CI from non-CI in NPC patients with late-delayed RTLI, and to assess their correlation with MoCA scores.

## 2 Methods

### 2.1 Study participants

This study received approval from the Institutional Review Board of the Sun Yat-Sen Memorial Hospital, Sun Yat-Sen University (Approval No: SYSKY-2022-271-03), with all enrolled subjects submitting signed informed consent forms. From January 2022 to July 2024, a total of 98 NPC patients with RTLI were recruited to undergo NODDI MRI. Eligibility for participation required meeting the following conditions: (1) histopathological confirmation of NPC obtained through nasopharyngoscopy; (2) a history of radiotherapy for NPC with imaging evidence of RTLI on conventional MRI as previously described (Wang et al., 2010); (3) no history of other malignancies; (4) no diagnosed central nervous system disorders, including Alzheimer’s disease or cerebrovascular disease; and (5) no documented history of hypertension, diabetes mellitus, nephrotic syndrome, or other major chronic illnesses. The exclusion criteria were as follows: (1) inability to complete the NODDI MRI examination due to poor clinical status and (2) poor image quality due to motion artifacts. Following the application of these criteria, the final analytical cohort comprised 90 patients (64 male, 26 female), with a mean age of 53.2 years (range: 29–76 years).

### 2.2 MRI examination

All patients underwent conventional MRI and NODDI on a 3.0 T scanner (MAGNETOM Skyra; Siemens Healthcare, Erlangen, Germany) using a 20-channel head/neck coil. The conventional imaging protocol comprised T2WI and T1WI sequences, alongside DWI acquired with b-values set at 0 and 1,000 s/mm^2^. NODDI data were acquired using an axial diffusion spectrum imaging (DSI) sequence with a 3D Cartesian q-space grid (grid radius r = 3). The acquisition comprised 99 diffusion-encoding directions, including one non-diffusion-weighted reference (b = 0 s/mm^2^) and 98 diffusion-weighted volumes sampled across 8 distinct b-value shells naturally distributed by the q-space grid geometry: 350 (6 directions), 650 (12), 1,000 (8), 1,350 (6), 1,650 (24), 2000 (24), 2,700 (12), and 3000 (6) s/mm^2^. The complete diffusion-encoding scheme, including the distribution of gradient directions across the diffusion shells, was described previously (Chiang et al., 2014). The total acquisition time for the DSI sequence was 6 min and 20 s. Following the intravenous injection of 0.1 mmol/kg gadobutrol contrast agent, axial contrast-enhanced T1WI was conducted. The specific technical parameters for each magnetic resonance sequence are comprehensively provided in Supplementary Table S1.

### 2.3 dMRI data analysis

All dMRI datasets were analyzed utilizing the NeuDiLab software (MRStation, V2, Zhongying, Chengdu, China), which is developed based on the open-source DIPY framework (Diffusion Imaging in Python, http://nipy.org/dipy). The preprocessing pipeline included skull stripping and eddy current correction. Quantitative parameters were subsequently derived from the following two diffusion models:

The ADC value was calculated with the following equation (Dixon, 1988):

$$S_{b}=S_{0}\times \exp(-b\times \mathrm{ADC})$$

where *S_b_* is the signal intensity at a given b value and *S_0_* is the signal intensity for b = 0 s/mm^2^.

The NODDI model quantifies tissue microstructure through three parameters: the ISOV, representing the free water compartment; the ICVF, reflecting diffusion restricted within neurites; and the ODI, describing the orientation dispersion of fibers within a voxel. ISOVF, ICVF, and ODI are calculated using the following equation (Kodiweera et al., 2016):

$$S=(1-\text{ISOVF})((1-\text{ICVF})\times S_{\text{extra}}+\text{ICVF}\times S_{\text{intra}}(\mathrm{ODI})+\text{ISOVF}\times S_{\mathrm{iso}})$$

where *S_intra_* denotes the signal contribution from the intracellular compartment, *S_extra_* represents the signal arising from the extracellular space, and *S_iso_* corresponds to the signal from the isotropic free-water compartment.

All diffusion parametric maps (ICVF, ODI, ISOVF, and ADC) and post-contrast axial T1-weighted images were coregistered to the T2-weighted reference images. For the diffusion parametric maps, an indirect registration was performed: first, the corresponding b = 0 images (which provide better contrast for registration) were rigidly registered to the T2-weighted images using Elastix (http://elastix.isi.uu.nl/); subsequently, the same transformation was applied to each diffusion parametric map to align them to the T2-weighted images. Regions of interest (ROIs) were manually delineated on T2-weighted hyperintense white matter areas by two independent neuroradiologists (QW and JL, with 4 and 5 years of experience in neuroradiology, respectively) using ITK-SNAP software (http://www.itksnap.org/pmwiki/pmwiki.php). Each radiologist performed their assessment without access to the MoCA scores. To minimize confounding effects, ROIs were carefully drawn to exclude areas of necrosis, cystic change, hemorrhage, or contrast-enhancing regions. In cases of bilateral lesions, ROIs were placed on the largest cross-section per side and the resulting parameter values were averaged; for unilateral lesions, a single ROI was drawn on the largest cross-section. The defined ROIs were then transferred to the co-registered NODDI parametric maps (ICVF, ODI, and ISOVF) and ADC maps to extract the mean values of each metric. Inter-rater agreement for ROI delineation was assessed using the Dice coefficient (Otani et al., 2025). Intraclass correlation coefficients (ICC) were used to assess the consistency of NODDI parameters (ICVF, ISOVF, and ODI) derived from the two independent ROI delineations. ICC values were interpreted as follows: <0.5 poor, 0.5–0.75 moderate, 0.75–0.90 good, and >0.90 excellent reliability.

### 2.4 Cognitive functional assessment

Cognitive function was assessed within three days after the MR examination using the MoCA, which was administered to all patients by a professional neurologist (XP, with 10 years of experience). The total MoCA score (range: 0–30) provides a global measure of cognitive performance. In accordance with standard scoring guidelines (Nasreddine et al., 2005), one additional point was granted to patients with fewer than 12 years of education or those over 65 years of age. A final score below 26 was used to define CI (Wu et al., 2020).

### 2.5 Statistical analysis

In the estimation of sample size for the NPC patients with RTLI, at least 48 cases (36 patients with CI and 12 patients without CI) were required in each dataset. The required sample size for each dataset was calculated based on the following parameters: a statistical power of 0.9, a two-sided significance level set at 0.05, and an alternative hypothesis assuming an AUC of 0.800 against a null hypothesis AUC of 0.500, with an assumed ratio of positive to negative cases of 3:1.

The normality of data distributions was evaluated with the Shapiro–Wilk test, while the homogeneity of variances across groups was examined using Levene’s test. Continuous variables are presented as mean ± standard deviation or median (interquartile range), and categorical variables as numbers (percentages). Group comparisons of NODDI metrics, ADC values, and clinical variables between patients with and without CI were conducted using appropriate statistical methods, including the independent t-test, Mann–Whitney U test, or chi-square test. The discriminative performance of parameters was subsequently assessed using receiver operating characteristic (ROC) curve analysis to evaluate the ability of significant diffusion metrics in discriminating CI patients from non-CI individuals. The discriminative accuracy was quantified by reporting the area under the ROC curve, accompanied by its two-sided 95% confidence interval. The optimal cutoff value was determined based on the Youden index. The ability to differentiate between patients with and without CI was evaluated using several standard metrics: sensitivity, specificity, positive predictive value (PPV), and negative predictive value (NPV). AUCs between different metrics were compared using DeLong’s test, while sensitivities, specificities, and accuracies were compared using the McNemar test. Correlations between NODDI-derived metrics (or ADC values) and MoCA scores were examined using Spearman’s correlation analysis. Multivariable linear regression analysis was performed to identify independent predictors of the MoCA score, with NODDI parameters (ICVF, ISOVF, and ODI) and education level (categorized as <6 years, 6–12 years, and >12 years, with the lowest level as reference) entered as independent variables. A two-tailed *p*-value < 0.05 was considered statistically significant. All statistical analyses and visualizations were performed using R software (version 3.3.3).

## 3 Results

### 3.1 Participant demographic and clinical characteristics

A total of 90 NPC patients with RTLI were included, consisting of 66 with CI and 24 without CI. The demographic and clinical characteristics of the participants are shown in Table 1. No statistically significant differences were observed between the two groups in age, sex, lesion distribution, or interval between radiotherapy completion and MRI (all *p* > 0.05). However, education level differed significantly between the two groups (*p* = 0.001).

**Table 1:** Demographic and clinical characteristics of patients with and without cognitive impairment.

Characteristic	Patients with cognitive impairment (*n* = 66)	Patients without cognitive impairment (*n* = 24)	*p*-value
Age			
Mean ± SD, years	51 ± 10	48 ± 10	0.280^Δ^
Range, years	29–76	35–72	
Gender (%)			>0.990^◊^
Male	47 (71)	17 (71)	
Female	19 (29)	7 (29)	
Education level (%)			0.001^◊^
<6y	20 (30)	3 (13)	
6–12y	28 (42)	8 (33)	
>12y	18 (27)	13 (54)	
Lesion distribution (%)			1.000^◊^
Unilateral	40 (61)	15 (62)	
Bilateral	26 (39)	9 (38)	
Time from completion of radiotherapy to MRI acquisition, years	4.00 (3.00, 9.50)	3.25 (2.75, 5.25)	0.133^●^

Unless otherwise stated, data are number (percentage).

Data was based on 90 brain lesions (with cognitive impairment, *n* = 66; without cognitive impairment, *n* = 24).

Δ: The independent samples t-test.

●: The Mean-Whitney U test.

◊: The Pearson’s χ^2^ test.

### 3.2 Comparison of NODDI parameters and ADC value between patients with and without CI

**Figure 1:**
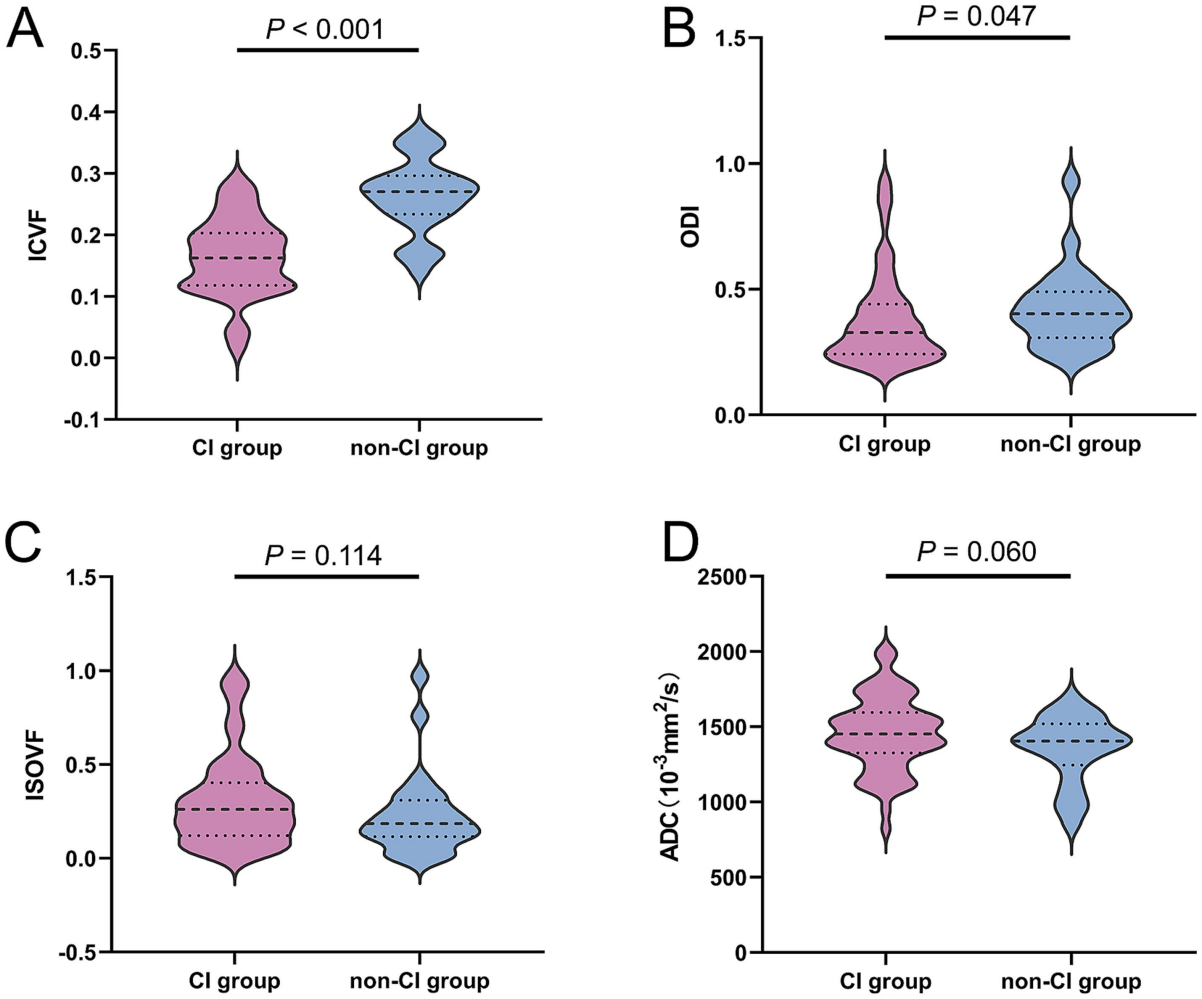
Differences in NODDI metrics and ADC value between patients with and without CI. CI group: patients with cognitive impairment; non-CI group: patients without cognitive impairment. **(A)** Comparison of ICVF between the two groups. **(B)** Comparison of ODI between the two groups. **(C)** Comparison of ISOVF between the two groups. **(D)** Comparison of ADC between the two groups.

**Figure 2:**
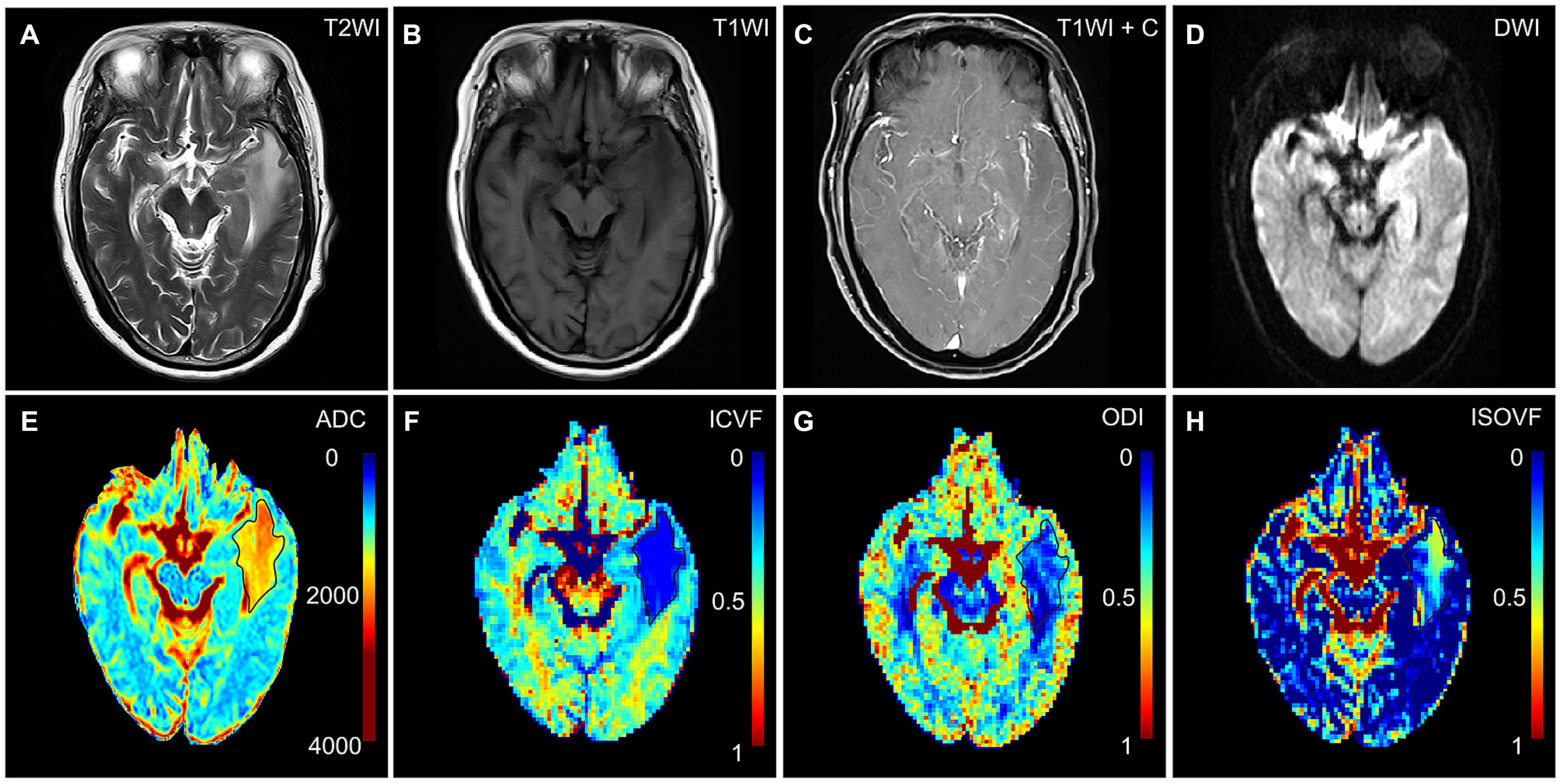
A representative case is a 53-year-old female NPC patient who presented with cognitive impairment, as indicated by a MoCA score of 11. **(A)** T2W images, **(B)** T1W images, **(C)** post-contrast T1W images, and **(D)** diffusion-weighted images. Pseudocolorful maps of ADC **(E)**, ICVF **(F)**, ODI **(G)**, and ISOVF **(H)**.

**Figure 3:**
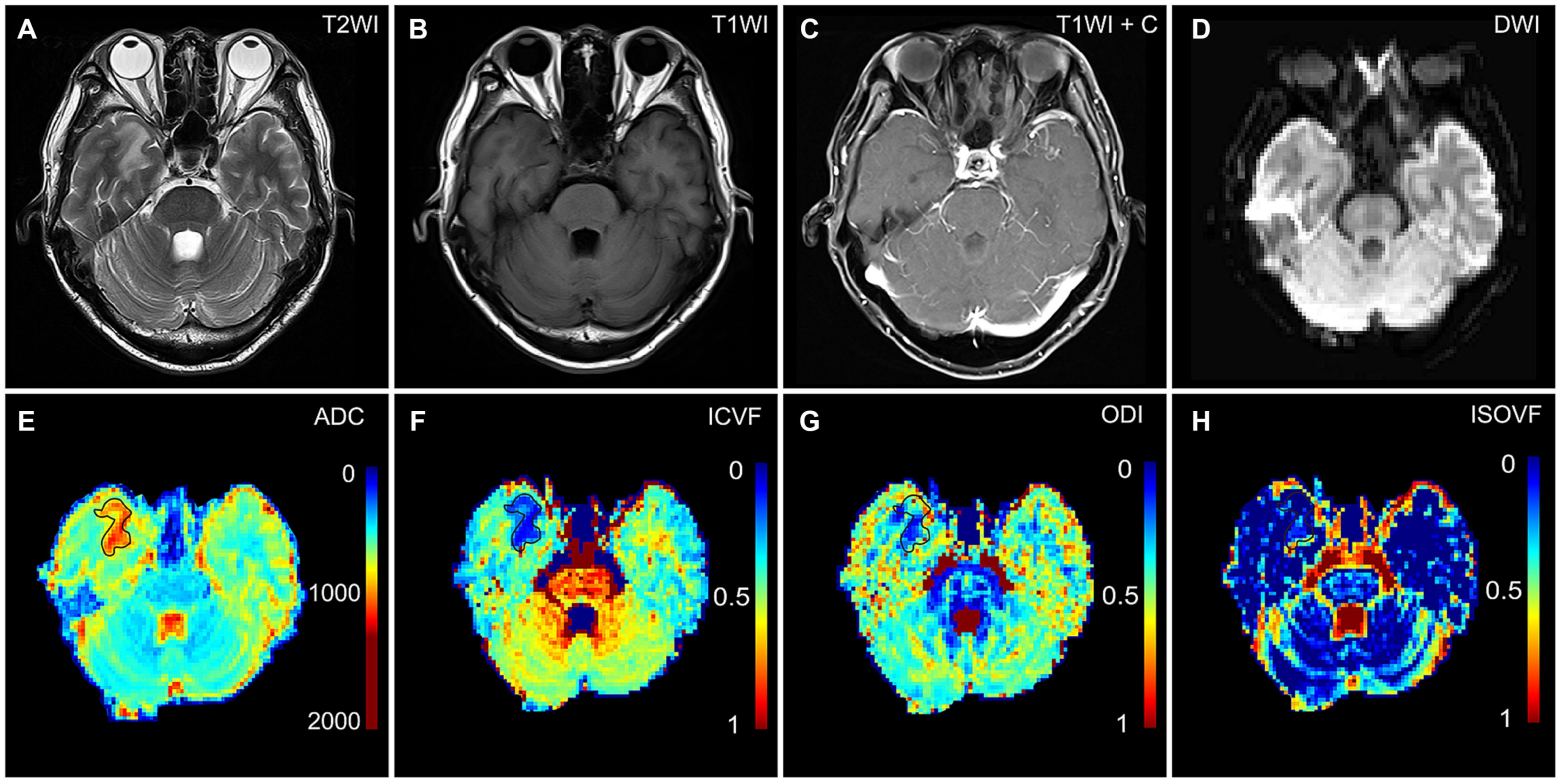
A representative case is a 54-year-old male NPC patient who presented without cognitive impairment, as indicated by a MoCA score of 27. **(A)** T2W images, **(B)** T1W images, **(C)** post-contrast T1W images, and **(D)** diffusion-weighted images. Pseudocolorful maps of ADC **(E)**, ICVF **(F)**, ODI **(G)**, and NODDI_ISOVF **(H)**.

The intraclass correlation coefficients for ADC, ICVF, ISOVF, and ODI were 0.968, 0.989, 0.998, and 0.999, respectively. The Dice coefficient for the ROIs drawn by the two readers was 0.866 ± 0.048. Mean values of NODDI parameters and ADC of the two groups are presented in Supplementary Table S2. Compared to the non-CI group, the CI group exhibited significantly lower ICVF and ODI values (*p* < 0.001 and *p* = 0.047, respectively) (Figures 1A,B). In contrast, no significant differences were found in ISOVF and ADC between the two groups (*p* > 0.05) (Figures 1C,D). Two representative cases of NPC patients with RTLI, one with CI and one without, are presented in Figures 2, 3, respectively.

### 3.3 Performances of NODDI parameters in differentiating between patients with and without CI

**Figure 4:**
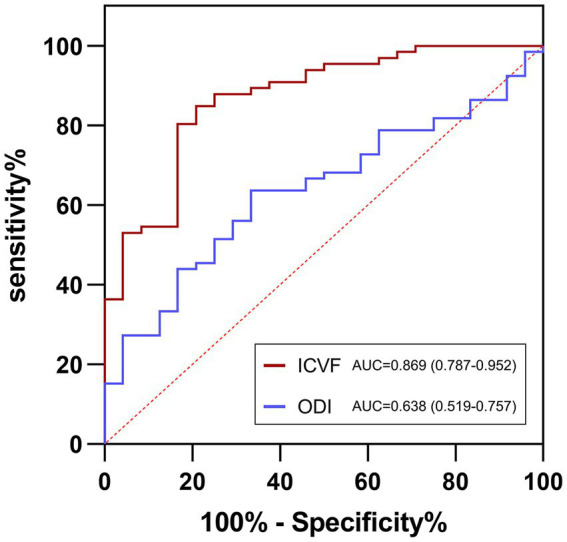
The receiver operating characteristic (ROC) curves of NODDI_ICVF and NODDI_ODI for discriminating between patients with and without CI.

The ROC analyses of the NODDI metrics are shown in Supplementary Table S3 and in Figure 4. ICVF demonstrated significantly better performance than ODI in differentiating patients with and without CI, with an AUC of 0.869 versus 0.638 (*p* < 0.001). Furthermore, ICVF showed higher sensitivity (84.8% vs. 63.6%, *p* < 0.05) and overall accuracy (83.3% vs. 64.4%, *p* < 0.05) compared to ODI, while no significant differences were found in specificity (79.2% vs. 66.7%, *p* > 0.05). Multivariable linear regression analysis showed that ICVF was significantly and independently associated with MoCA score (*β* = 0.766, *p* < 0.001). In contrast, ISOVF, ODI, and education level were not significantly associated with MoCA scores in the multivariable model (all *p* > 0.05). Detailed results are presented in Supplementary Table S4.

### 3.4 Correlation between NODDI metrics, ADC value, and MoCA scores

**Figure 5:**
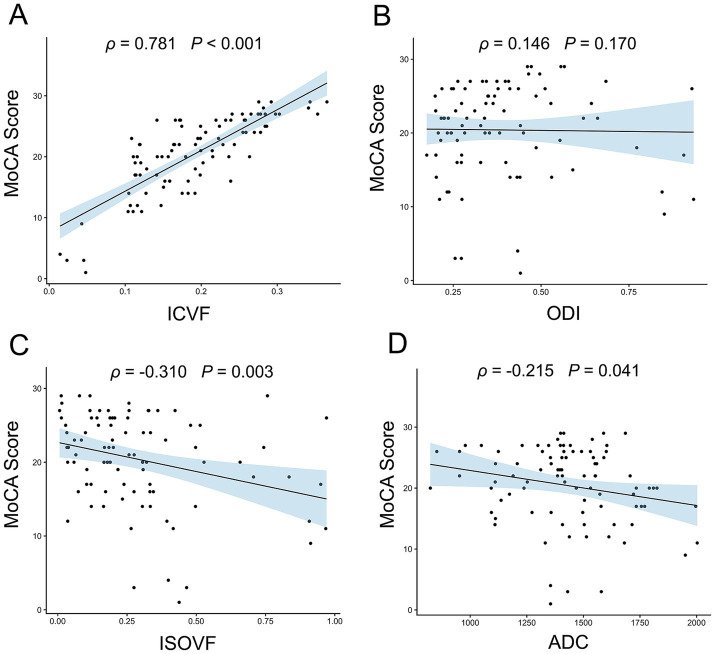
Scatter plot showing the correlation between NODDI-derived metrics or ADC and MoCA scores. The trend line represents the linear regression fit. **(A)** Scatter plot showing the correlation between MoCA score and ICVF. **(B)** Scatter plot showing the correlation between MoCA score and ODI. **(C)** Scatter plot showing the correlation between MoCA score and ISOVF. **(D)** Scatter plot showing the correlation between MoCA score and ADC.

The correlation coefficients between MoCA score and NODDI metrics are summarized in Figure 5. ICVF showed a strong positive correlation with MoCA scores (*ρ* = 0.781, *p* < 0.001), whereas ISOVF (*ρ* = −0.310, *p* = 0.003) and ADC (*ρ* = −0.215, *p* = 0.041) showed weak negative correlations. No significant correlations were observed between MoCA scores and ODI (*p* > 0.05).

## 4 Discussion

Our study demonstrated that NODDI-derived ICVF and ODI were markedly reduced in NPC patients with late-delayed RTLI and CI than in those with RTLI, but without CI. The ICVF exhibited high performance in differentiating CI from non-CI (AUC = 0.869) and strongly correlated with the MoCA scores (*ρ* = 0.781). These findings highlight ICVF as a potential clinically relevant imaging biomarker associated with CI and evaluating the severity of RTLI in NPC patients.

RTLI leads to white matter microstructural damage, which constitutes a key pathological substrate of CI (Piao et al., 2015; Salehi et al., 2022; Hosp et al., 2024). In the present study, NPC patients with CI exhibited significantly lower ICVF and ODI values in the affected white matter regions than those without CI. These two NODDI-derived parameters capture distinct yet complementary aspects of radiation-induced white matter pathology. ICVF quantifies the volume fraction occupied by neurites, serving as a direct marker of neurite density. A reduction in the ICVF reflects axonal degeneration or dendritic loss (Shao et al., 2021). In contrast, ODI characterizes the angular variation of neurite orientations within a voxel. A decrease in ODI indicates a reduction in the complexity of neural architecture, often resulting from the loss of crossing fibers or the progressive simplification of tissue structure due to demyelination and gliosis (Thaker et al., 2023). Consequently, the resulting disruption of neuronal signaling leads to the occurrence of CI (Zheng et al., 2022; Araki et al., 2024). Recently, Zhou et al. applied the NODDI model to investigate microstructural brain alterations following whole-brain radiotherapy in patients with brain metastases (Zhou et al., 2024). Their study revealed microstructural damage in multiple brain regions after treatment. NODDI-derived features predicted cognitive changes that correlated significantly with measured changes at one month (R = 0.573) and three months (R = 0.687). These findings suggest that NODDI-derived features were associated with cognitive changes following whole-brain radiotherapy. Our findings are also consistent with previous reports on other neurological conditions. For instance, individuals with color discrimination deficits—a function linked to cognitive decline—also showed decreased ICVF and ODI compared with controls (Zhang et al., 2025). Similarly, Preziosa et al. observed that multiple sclerosis patients with CI exhibited lower ICVF and ODI in the white matter than those with preserved cognition (Preziosa et al., 2023). ICVF demonstrated superior performance to ODI in differentiating CI from non-CI, and its correlation with MoCA score further supports ICVF as a potential biomarker for cognitive function in RTLI patients. This suggests that neurite density, as measured by ICVF, may be more directly associated with cognitive status than fiber organization. Supporting this notion, Ke Ning et al. found that neurite density index (NDI) provided superior performance over ODI in differentiating CI in patients with obstructive sleep apnea hypopnea syndrome (Ning et al., 2024). Similarly, Fu et al. reported that (Fu et al., 2020) a significant correlation was observed between the NDI values in Alzheimer’s Disease patients and their corresponding scores on the MoCA (r = 0.647, *p* < 0.001). In the present study, although ODI differed significantly between the CI and non-CI groups, it did not show a significant correlation with MoCA scores. This may be because cognitive status is more directly linked to loss of neurite density and disruption of structural connectivity than to changes in fiber orientation alone. ISOVF showed only a weak negative correlation with MoCA scores (*ρ* = −0.310). Although ISOVF reflects the isotropic free water compartment and can be elevated in vasogenic edema or neuroinflammation, late-delayed RTLI is predominantly characterized by chronic neurite loss and gliosis rather than acute edema (Zheng et al., 2022). Thus, ISOVF changes are likely secondary and less directly associated with cognitive function, accounting for its relatively weak correlation with MoCA scores. In summary, NODDI-derived ICVF has shown considerable potential as an emerging neuroimaging biomarker associated with CI in RTLI patients, and its microstructural relevance to cognitive function suggests potential utility in other neurological disorders involving neurite damage.

Conventional DWI, quantified by ADC, provides a non-specific measure of overall water mobility for assessing the tissue microstructure. In this study, ADC values did not demonstrate a statistically significant distinction between subjects exhibiting cognitive impairment (*p* > 0.05). This finding is consistent with a report by H-Z Wang et al., who observed negligible ADC differences between post-radiotherapy NPC patients and healthy controls (Wang et al., 2012). These results highlight the limited utility of ADC for evaluating radiation-induced cerebral damage. This is due to the fact that ADC represents a composite measure of water diffusion that is influenced by multiple coexisting tissue factors (Xu et al., 2020). In the late-delayed stage of RTLI, several coexisting pathological processes exert opposing effects on ADC. Demyelination and axonal loss tend to increase water diffusivity, whereas reactive gliosis and cellular debris may restrict water movement, such that the opposing effects cancel each other out. Additionally, the presence of vasogenic edema (increasing ADC) alongside microglial activation and fibrosis (decreasing ADC) further complicates the ADC signal. These counteracting influences may explain why ADC fails to discriminate CI from non-CI patients. In contrast, NODDI is a biophysically grounded diffusion model that decomposes the diffusion signal into three distinct compartments: intracellular (reflecting neurite density), orientation dispersion, and isotropic (representing free water) (Zhang et al., 2012). By providing specific measures of these tissue characteristics, NODDI enables more precise characterization of microstructural alterations associated with CI. Consequently, NODDI represents a more advanced approach associated with radiation-induced cognitive deficits than conventional DWI.

This study acknowledges several methodological constraints. The cohort size was limited, and all subjects were enrolled prospectively from a single medical center. Although the actual number of enrolled patients in both the CI and non-CI groups exceeded the estimated sample sizes required for statistical power (36 and 12, respectively), the generalizability of our findings should be further validated in a larger, multicenter cohort. Secondly, the ROI analysis in this study was confined to T2-hyperintense white matter in the temporal lobes, thereby excluding other radiation-induced lesions such as T1W contrast-enhancing areas and regions of cystic transformation. This approach is justified by literature emphasizing the association between T2W white matter hyperintensities in post-radiotherapy and cognitive decline (Douw et al., 2009). Future research should expand the analysis to include cortical and subcortical gray matter, which may potentially enable a more thorough elucidation of the structural underpinnings associated with cognitive impairment. Additionally, detailed information regarding radiotherapy regimens, including total radiation dose, fractionation schedule, and treatment field design, was not available for analysis in the present study due to the prolonged disease course and the fact that many patients received their initial treatment at other institutions. Future studies with comprehensive treatment data are warranted to investigate how specific radiotherapy parameters may influence the progression of microstructural damage and subsequent cognitive outcomes, which could provide valuable insights for optimizing treatment strategies to minimize neurocognitive sequelae. Fourth, the relatively long acquisition time of NODDI may increase motion artifact risk in CI patients. Emerging acceleration techniques, including compressed sensing (Radhakrishnan et al., 2024) and deep learning algorithms (Xiao et al., 2025), could help mitigate this limitation in future studies.

## 5 Conclusion

In conclusion, this study identified NODDI-derived ICVF as an potential imaging biomarker associated with CI in NPC patients with late-delayed RTLI, outperforming ADC in terms of discriminative ability. Its strong correlation with MoCA scores suggests that the ICVF may serve as a correlate of cognitive status. As a noninvasive advanced diffusion technique, NODDI holds potential for optimizing clinical management strategies in RTLI.

## Data Availability

The raw data supporting the conclusions of this article will be made available by the corresponding author upon reasonable request, without undue reservation.
