# Supplementary material for: Association of neurite orientation dispersion and density imaging with cognitive impairment in nasopharyngeal carcinoma patients with radiation-induced temporal lobe injury
Source: Front Hum Neurosci. 2026 Jun 1;20:1809205. doi: 10.3389/fnhum.2026.1809205 (PMC13265276; doi:10.3389/fnhum.2026.1809205)
Supplement: Supplementary file 1 [file Table_1.docx]

Supplementary Material

# Supplementary Tables

**Table S1 Imaging sequences and acquisition parameters of structural and diffusion MRI**

| **Sequence** | **TR (ms)** | **TE (ms)** | **FOV (mm)** | **Matrix** | **Slice thickness (mm)** | **Slice gap (mm)** | **b value (s/mm^2^)** | **Acquisition time** |
| --- | --- | --- | --- | --- | --- | --- | --- | --- |
| **T1WI** | 2000 | 9 | 220×220 | 320×240 | 5 | 1 | - | 1 min 20 s |
| **T2WI** | 4500 | 96 | 220×220 | 320×240 | 5 | 1 | - | 1 min 39 s |
| **T1W+C** | 180 | 5 | 220×220 | 320×240 | 5 | 1 | - | 2 min 20 s |
| **DWI** | 4700 | 85 | 220×220 | 130×130 | 5 | 1 | 0/1000 | 34 s |
| **DSI** | 3600 | 109 | 220×220 | 110×110 | 4 | 0.8 | 0-3000 | 6 min 20 s |

b values for DSI includes 0, 350, 650, 1000, 1350, 1650, 2000, 2700, 3000 s/mm^2^.

T1WI, T1-weighted imaging; T2WI, T2-weighted imaging; T1+C, post-contrast T1-weighted imaging; DWI, diffusion-weighted imaging; DSI, diffusion spectrum imaging; TR, repetition time; TE, echo time; FOV, field of view.

**Table S2 NODDI metrics and ADC value in patients with and without cognitive impairment**

| **Metrics** | **Patients with CI**  **(*n* = 66)** | **Patients without CI**  **(*n* = 24)** | ***P* Value** |
| --- | --- | --- | --- |
| **NODDI_ICVF** | 0.163 (0.118,0.203) | 0.270 (0.234,0.296) | <.001* |
| **NODDI_ISOVF** | 0.261 (0.121,0.404) | 0.186 (0.116,0.309) | .114 |
| **NODDI_ODI** | 0.328 (0.242,0.441) | 0.403 (0.307,0.490) | .047* |
| **ADC** **(10^−3^ mm^2^/s)** | 1459.69 ± 245.83 | 1354.71 ± 221.19 | .060 |

* *P* < 0.05 indicates statistical significance.

**Table S3 Performance of NODDI-derived metrics in discriminating patients with and without cognitive impairment**

| **NODDI Metrics** | **AUC** | **Cutoff value** | **Sensitivity (%)** | **Specificity (%)** | **Accuracy (%)** | **PPV (%)** | **NPV (%)** |
| --- | --- | --- | --- | --- | --- | --- | --- |
| **NODDI_ICVF** | 0.869  [0.787, 0.952] | 0.230 | 84.8 (56/66)  [73.9, 92.5] | 79.2 (19/24)  [57.9, 92.9] | 83.3 (75/90)  [74.0, 90.4] | 91.8 (56/61)  [81.9, 97.3] | 65.5 (19/29)  [45.7, 82.1] |
| **NODDI_ODI** | 0.638  [0.519, 0.757] | 0.371 | 63.6 (42/66)  [50.9, 75.1] | 66.7 (16/24)  [44.7, 84.4] | 64.4 (58/90)  [53.7, 74.3] | 84.0 (42/50)  [70.9, 92.8] | 40.0 (16/40)  [24.9, 56.7] |

AUC, area under the curve; PPV, positive predictive value; NPV, negative predictive value.

Data in the brackets indicate the numerator and denominator of the obtained parameters.

Data in the square brackets indicate the 95% confidence intervals.

**Table S4 Multivariable linear regression analysis of NODDI metrics and education level with MoCA score**

| **Variables** | **B (95% CI)** | ***β*** | ***P* Value** |
| --- | --- | --- | --- |
| **Education Level** |  |  |  |
| <6y | 0 (Ref.) | - | - |
| 6-12y | 0.948 (-1.784-3.680) | 0.075 | 0.492 |
| >12y | 1.831 (-0.920-4.582) | 0.144 | 0.189 |
| **NODDI_ICVF** | 64.160 (52.332-75.988) | 0.766 | < 0.001* |
| **NODDI_ISOVF** | -2.543 (-7.312-2.227) | -0.096 | 0.292 |
| **NODDI_ODI** | -1.760 (-8.204-4.684) | -0.049 | 0.588 |

* *P* < 0.05 indicates statistical significance.

Ref. = Reference group; B = unstandardized regression coefficient; *β* = standardized regression coefficient; CI = confidence interval.
